# Management and Prognosis of Anti-MDA5 Dermatomyositis: Insights from a National Multicenter Cohort

**DOI:** 10.3390/biomedicines14030709

**Published:** 2026-03-19

**Authors:** Sándor Mogyoróssy, Zoltán Griger, Tünde Tarr, Éva Zöld, György Pfliegler, Boglárka Csilla Brúgós, György Nagy, Károly Zsolt Mangel, Gábor Kumánovics, Rita Bakai, László Kovács, Adrienn Rideg, Edit Nagy, Orsolya Farkas, Gábor Nagy, Péter Antal-Szalmás, Gabriella Szűcs, Szilvia Szamosi, Zoltán Szekanecz, Éva Rákóczi, Levente Bodoki

**Affiliations:** 1 Department of Rheumatology, Faculty of Medicine, University of Debrecen, 4032 Debrecen, Hungary; sandormogyorossydr@gmail.com (S.M.); szucs.gabriella@med.unideb.hu (G.S.); szamosi.szilvia@med.unideb.hu (S.S.); szekanecz.zoltan@med.unideb.hu (Z.S.); eva.rakoczi@gmail.com (É.R.); 2 Doctoral School of Medical Sciences, University of Debrecen, 4032 Debrecen, Hungary; 3 Division of Clinical Immunology, Faculty of Medicine, University of Debrecen, 4032 Debrecen, Hungary; grigerz@googlemail.com (Z.G.); tarr.tunde@med.unideb.hu (T.T.); zold_eva@yahoo.com (É.Z.); 4 Centre of Rare Diseases, Department of Internal Medicine, University of Debrecen, 4032 Debrecen, Hungary; g.pfliegler@gmail.com (G.P.); brugosb@gmail.com (B.C.B.); 5 Department of Rheumatology and Immunology, Semmelweis University, 1023 Budapest, Hungary; gyorgyngy@gmail.com (G.N.); mangelzsolt@gmail.com (K.Z.M.); 6 Heart and Vascular Center, Semmelweis University, 1122 Budapest, Hungary; 7 Department of Genetics, Cell- and Immunobiology, Semmelweis University, 1089 Budapest, Hungary; 8 Department of Rheumatology and Immunology, Faculty of Medicine, University of Pécs, 7632 Pécs, Hungary; kumanovics.gabor@pte.hu (G.K.); bakairita93@gmail.com (R.B.); 9 Department of Rheumatology and Immunology, Albert Szent-Györgyi Medical School, University of Szeged, 6725 Szeged, Hungary; kovacs.laszlo@med.u-szeged.hu (L.K.); adrienn.rideg21@gmail.com (A.R.); 10 Division of Radiology and Imaging Science, Department of Medical Imaging, Faculty of Medicine, University of Debrecen, 4032 Debrecen, Hungary; edoedo0624@gmail.com; 11 Department of Diagnostic Imaging, University of Pécs, 7623 Pécs, Hungary; farkas.orsolya@pte.hu; 12 Department of Laboratory Medicine, Faculty of Medicine, University of Debrecen, 4032 Debrecen, Hungaryantalszp@med.unideb.hu (P.A.-S.)

**Keywords:** anti-MDA5 antibody, dermatomyositis, interstitial lung disease (ILD), anti-Ro52, rapidly progressive ILD, prognosis

## Abstract

**Background**: Anti-melanoma differentiation-associated gene 5 (anti-MDA5) positive dermatomyositis is a distinct subset of idiopathic inflammatory myopathies (IIMs), often associated with unique cutaneous features and interstitial lung disease (ILD). While East Asian cohorts frequently report high mortality due to rapidly progressive ILD (RP-ILD), data regarding Central and Eastern European populations remain scarce. **Methods**: We conducted a retrospective multicenter study of anti-MDA5 positive Caucasian patients managed at four Hungarian rheumatology centers between 2020 and 2025. Demographic, clinical, serological, and radiological data were analyzed. Antibody profiling was performed using a standardized 16-antigen immunoblot assay. **Results**: Anti-MDA5 positivity was confirmed in 24 out of 742 patients (3.23%) treated in the four centers. The median age at diagnosis was 49.5 years (range: 24–81). Classic dermatomyositis was the predominant clinical phenotype (75%), followed by clinically amyopathic dermatomyositis (CADM) (12.5%) and polymyositis (12.5%). ILD was identified in 58.3% of patients, presenting with organizing pneumonia (OP), non-specific interstitial pneumonia (NSIP), and usual interstitial pneumonia (UIP) patterns. At diagnosis, median creatine kinase (CK) (193.5 U/L) and C-reactive protein (CRP) (4.24 mg/L) levels remained low even in the ILD group, whereas lactate dehydrogenase (LDH) was elevated in 91.7% of the cohort. Anti-Ro52 positivity (45.8% overall) emerged as a notable predictor of ILD (odds ratio [OR]: 22.5, 95% confidence interval [CI]: 2.10–240.48; *p* = 0.0045), being present in 71.4% of affected patients. RP-ILD occurred in two patients (8.3%). Therapeutic management followed an early, aggressive strategy, frequently utilizing cyclophosphamide (45.8%) and methotrexate (37.5%), with Janus kinase (JAK) inhibitors or rituximab employed in refractory cases. Overall disease-specific survival was 100% during the study period (median follow-up: 72.0 months); no mortality was directly attributable to IIM-related complications. **Conclusions**: Our study demonstrates that anti-MDA5 positive dermatomyositis in a Hungarian cohort is characterized by heterogeneous manifestations and a significant association between anti-Ro52 and ILD. The observed dissociation between low CK/CRP and elevated LDH underscores the necessity for a high index of suspicion, with LDH serving as a superior marker for disease activity. While ILD presents a significant risk, early and intensive multi-modal intervention may yield superior survival outcomes in European patients compared to the historical mortality rates reported in Asian cohorts.

## 1. Introduction

The discovery of new autoantigen systems associated with idiopathic inflammatory myopathies (IIMs) in adults and children has had a significant impact on the diagnosis, classification, and treatment of this group of diseases [1]. Myositis-specific antibodies (MSAs) are associated with distinct clinical phenotypes and organ involvements, aiding in the stratification of patients.

Anti-melanoma differentiation-associated gene 5 (anti-MDA5) antibody (formerly called anti-CADM-140) is a distinct MSA identified in 2005 by Sato et al. [2,3]. Originally described in patients with clinically amyopathic dermatomyositis (CADM), anti-MDA5 is considered a dermatomyositis (DM)-specific autoantibody. The clinical presentation is unique and often includes minimal or absent muscle weakness (hypomyopathic phenotype), characteristic cutaneous features (such as skin ulcers, palmar papules, or mechanic’s hands), prominent articular involvement (often mimicking rheumatoid arthritis), and a high frequency of interstitial lung disease (ILD) [4,5].

The prognosis of anti-MDA5 positive myositis is historically considered poor due to the high prevalence of rapidly progressive ILD (RP-ILD), particularly in East Asian cohorts [6]. However, recent studies suggest a heterogeneous disease course, with conflicting data regarding mortality rates between Asian and Caucasian populations [7,8]. Emerging evidence posits that this divergence in clinical trajectory may be attributable to underlying immunogenetic differences, specifically the presence of distinct HLA profiles in Caucasian populations that appear to confer a more favorable prognosis compared to the genetic risk factors identified in Asian cohorts. Furthermore, the presence of co-occurring antibodies (e.g., anti-Ro52, also known as anti-TRIM21 /tripartite motif containing protein 21/) may further influence the clinical phenotype and outcomes, although these associations remain under investigated.

While data from Asia and Western Europe are accumulating, there is a scarcity of information regarding the characteristics of anti-MDA5 positive patients in Central and Eastern Europe. Addressing this gap is crucial, as distinct genetic backgrounds and differences in healthcare protocols may significantly influence disease phenotype and outcomes in this specific region compared to Asian or Western European populations.

The primary objective of this retrospective multicenter study was to characterize the clinical phenotype and disease course of anti-MDA5 positive dermatomyositis in a Central European Caucasian cohort, a population currently underrepresented in the literature. We sought to describe the prevalence and distribution of interstitial lung disease patterns and cutaneous manifestations, and to quantify the prognostic impact of co-occurring autoantibodies, specifically focusing on the association between anti-Ro52 and pulmonary involvement. Furthermore, the study aimed to evaluate real-world therapeutic strategies and survival outcomes, allowing for a comparative analysis to determine whether the high incidence of RP-ILD and mortality rates frequently reported in East Asian cohorts are applicable to this Central European population.

## 2. Materials and Methods

### 2.1. Patients and Study Design

This retrospective multicentric study reviewed the antibody profiles of myositis patients presenting between 1 January 2020 and 31 December 2025 in four selected Hungarian tertiary rheumatology centers. From this population, patients with confirmed anti-MDA5 positivity were selected for detailed analysis. All patients had a definitive diagnosis of myositis according to the Bohan and Peter or the ACR/EULAR classification criteria for IIMs. It is important to note that while the antibody testing was performed within the study period, in several cases, the specific anti-MDA5 positivity was identified retrospectively or during follow-up, years after the initial clinical diagnosis, once the extended myositis immunoblot became routinely available.

### 2.2. Data Collection

Pertinent clinical and laboratory information was collected retrospectively. Data sources included the local hospital information systems of the four participating centers and medical records. Clinical findings were categorized based on the presence or absence of muscular and extramuscular manifestations (Disease Activity Core Set Measures) in accordance with the International Myositis Assessment and Clinical Studies Group (IMACS) recommendations [9]. Detailed data regarding the patients’ antibody profiles and previous or current immunosuppressive therapies were also recorded.

### 2.3. Laboratory Assessments

In this study, anti-MDA5 positivity was detected using the same commercial immunoblot method at the local laboratory of each center. All four laboratories utilized the Autoimmune Inflammatory Myopathies 16 Ag (cat. No.: DL 1530-1601-4 G) immunoblot reagent kit from Euroimmun AG (Lübeck, Germany) for routine diagnostic purposes. This assay allows for the simultaneous determination of 16 autoantibodies: Mi-2α, Mi-2β, TIF1γ, MDA5, NXP2, SAE1, Ku, PM/Scl100, PM/Scl75, Jo-1, SRP, PL-7, PL-12, EJ, OJ, and Ro52. Blood samples were processed immediately after collection according to the manufacturer’s instructions, with serum samples diluted in a 1:101 ratio. Test strips were scanned and evaluated using the EuroLineScan software (version 3.4.37). Results were reported on a semi-quantitative scale. For the purpose of this study, only patients with weak (+), moderate (++), or strong (+++) positivity were included; borderline (+/−) results were considered negative and excluded from the analysis. The detection of anti-Ro52 antibody was performed using the same immunoblot. All participating laboratories are certified national diagnostic facilities that adhere to standardized, harmonized internal and external quality control procedures according to the manufacturer’s protocols.

### 2.4. Radiological Assessments

Patients were diagnosed with ILD if they met all the following three criteria: (1) respiratory symptoms including dry cough, wheezing and exertional dyspnea; (2) high-resolution CT (HRCT) scans showing ground-glass opacities, consolidations, reticulations and/or honeycombing; (3) lung function tests indicating restrictive impairments (total lung capacity and diffusing capacity of carbon monoxide [DLCO] < 80% of predicted) with the possible association of physical signs revealing Velcro crackles in the lung bases, and clubbing, excluding infection and drug-induced interstitial changes [10]. Patients were diagnosed with ILD strictly if they met all the following three criteria simultaneously. Borderline cases or those with only suspected clinical signs without definitive radiological and functional confirmation were excluded from the ILD group. RP-ILD was defined as experiencing any of the following four conditions within one month after DM symptom onset: (1) acute and progressive worsening of dyspnea requiring admission to the hospital or extra oxygen support; (2) progressive impairment of lung function, including over 10% drop in forced vital capacity (FVC) or more than 15% decrease in DLCO with reduced FVC; (3) radiological progression of HRCT (over 20% increase in interstitial abnormalities of the lung); (4) arterial blood gas abnormalities (results indicating respiratory failure or a drop in oxygen partial pressure exceeding 10 mmHg) [11]. Image interpretation, particularly the characterization of ILD patterns, was performed by radiologists with expertise in ILD diagnostics.

### 2.5. Statistical Analysis

Statistical analysis was performed using IBM SPSS Statistics version 27.0 (IBM Corp., Armonk, NY, USA) to describe the demographic and clinical characteristics of the cohort. Continuous variables were tested for normality; given the non-normal distribution of most laboratory parameters (e.g., CK, LDH levels), data are presented as medians with ranges (minimum-maximum) or interquartile ranges, rather than means. Categorical variables are expressed as frequencies and percentages. Comparisons between subgroups (e.g., ILD vs. non-ILD) were performed using the Mann–Whitney U test for continuous variables and Fisher’s exact test for categorical variables, due to the relatively small sample size. To quantify the strength of associations, odds ratios (OR) with 95% confidence intervals (CI) were calculated. A *p*-value of <0.05 was considered statistically significant.

### 2.6. Ethical Considerations

The study was approved by the Hungarian Scientific Research Council Ethical Committee (approval No. BM/11321-3/2023/EKU). The study was conducted in accordance with the Declaration of Helsinki and its amendments.

## 3. Results

### 3.1. Demographic Characteristics and Diagnosis

Anti-MDA5 positivity was confirmed in 24 patients out of a total of 742 patients treated in the four centers. All 24 patients were Caucasian. The median age at diagnosis was 49.5 years (range: 24–81 years). The female to male ratio was 1.67:1 (15 females and 9 males). According to the classical clinico-pathological subgroups, there were 18 DM patients (75%), 3 amyopathic DM (ADM) patients (12.5%) and 3 polymyositis (PM) patients (12.5%). Overlap syndromes were identified in four cases: three patients with DM-RA overlap and one patient with PM-SSc overlap. Additionally, one PM patient presented with a localized form of scleroderma (linear scleroderma). Notably, dermatomyositis was associated with primary immunodeficiency (PID) in two cases. A cancer-associated myositis patient experienced tumor recurrence (breast adenocarcinoma) on three occasions during the disease course.

### 3.2. Clinical Manifestations

The most important clinical and serological parameters are summarized in Table 1.

Muscle weakness was present in 21 patients (87.5%), affecting all patients except those in the ADM subgroup. Diagnostic procedures included muscle biopsy in 6 patients and electromyoneurography (EMNG) in 4 patients. The relatively low number of these invasive procedures reflects modern diagnostic paradigms, where the presence of highly specific autoantibodies (anti-MDA5) combined with pathognomonic cutaneous manifestations often establishes the diagnosis without necessitating muscle biopsy, particularly in hypomyopathic or amyopathic cases. Similarly, cutaneous symptoms typical of dermatomyositis (heliotrope rash, Gottron’s papules, Gottron’s sign, ulcerations, shawl sign, or V-sign) were observed in 21 patients (87.5%), occurring in all patients except those in the PM subgroup. Beyond the pathognomic cutaneous symptoms, mechanic’s hands were recorded in 4 patients (16.7%), and subcutaneous calcinosis was present in one case (4.2%).

Musculoskeletal involvement with arthritis or arthralgia affected 18 patients (75.0%). Systemic and organ manifestations included fever (n = 5, 20.8%), Raynaud’s phenomenon (n = 5, 20.8%, comprising 3 DM, 1 PM, and 1 PM-SSc overlap patients), dysphagia (n = 5, 20.8%), and myocarditis (n = 3, 12.5%).

**Figure 1:**
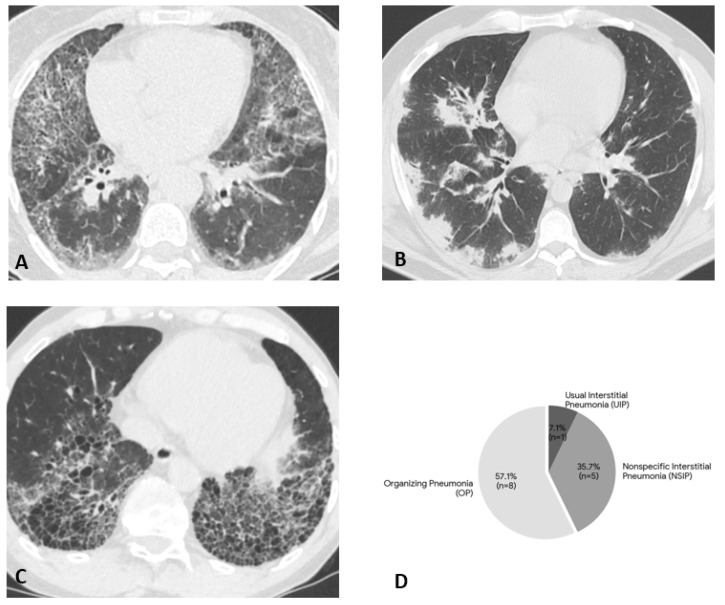
Radiographic manifestations of interstitial lung disease (ILD) in anti-MDA5 positive patients. (**A**–**C**): Representative High-Resolution Computed Tomography (HRCT) images showing different ILD patterns. (**A**) Nonspecific interstitial pneumonia (NSIP) pattern with extensive ground-glass opacity and fine reticulation. (**B**) Organizing pneumonia (OP) pattern with characteristic peripheral dominant consolidations and ground-glass opacities. (**C**) Usual interstitial pneumonia (UIP) pattern demonstrating honeycombing and traction bronchiectasis. (**D**) Pie chart illustrating the distribution of HRCT patterns among patients with lung involvement (n = 14). The OP pattern was the most frequent (n = 8, 57.1%), followed by NSIP (n = 5, 35.7%) and UIP (n = 1, 7.1%).

Interstitial lung disease (ILD) was identified in 14 patients (58.3%), showing variable radiological patterns: five patients with nonspecific interstitial pneumonia (NSIP), one with usual interstitial pneumonia (UIP), and eight with organizing pneumonia (OP) (Figure 1). In the ILD group, the female:male ratio was 1.33:1 (8 females and 6 males). A significant association was observed with anti-Ro52 positivity, which was present in 10 out of the 14 ILD patients (71.43%) compared to only 10% in the non-ILD group (OR: 22.5, 95% CI: 2.10–240.48; *p* = 0.0045). RP-ILD occurred in two patients with DM.

### 3.3. Laboratory Findings and Serological Profile

Laboratory investigations at the time of diagnosis showed that the median creatine kinase (CK) level was 193.5 U/L (range: 21–3463 U/L), with 17 patients (70.8%) presenting with a CK level below 500 U/L. The median lactate dehydrogenase (LDH) level was 377.0 U/L (range: 233–1538 U/L), and elevated LDH (>250 U/L) was present in 22 patients (91.7%). The median C-reactive protein (CRP) level was 4.24 mg/L (range: 0.66–33.0 mg/L); values above 5 mg/L were observed in 10 patients (41.7%).

All 24 patients were positive for anti-MDA5 antibodies. The most frequent co-occurring antibody was anti-Ro52, identified in 11 patients (45.8%). Other myositis-specific antibodies (MSAs) were detected in a few cases: anti-SAE in three patients (12.5%), anti-NXP2 in two patients (8.3%), and anti-SRP and anti-PL-7 in one patient (4.2%) each. Regarding myositis-associated antibodies (MAAs), anti-PM-Scl was present in three patients (12.5%), anti-Ku in three patients (12.5%), and anti-PL-12 in two patients (8.3%). No cases of anti-Mi-2, anti-TIF1-gamma, anti-Jo-1, anti-EJ, or anti-OJ positivity were identified. Antinuclear antibody (ANA) positivity was observed in 8 patients (33.3%). Recorded staining patterns included homogeneous (n = 3), granular/speckled (n = 4), and one case showing both homogeneous and granular patterns.

### 3.4. Therapy and Outcomes

**Figure 2:**
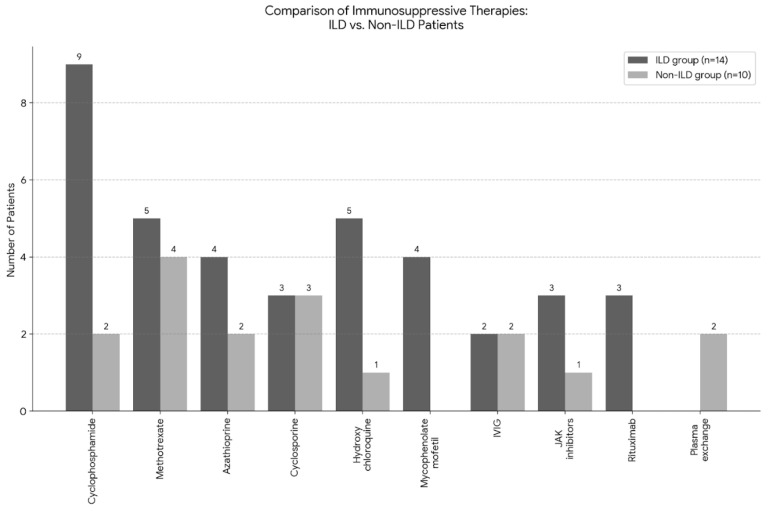
Comparison of immunosuppressive therapeutic strategies based on lung involvement. Grouped bar chart illustrating the utilization of immunosuppressive agents stratified by the presence of interstitial lung disease (ILD, n = 14) versus non-ILD phenotypes (n = 10). Systemic glucocorticoids were administered to all patients. Cyclophosphamide was predominantly utilized in the ILD group (n = 9, 64.3%) compared to the non-ILD group (n = 2, 20.0%), reflecting a risk-stratified, aggressive therapeutic approach for pulmonary manifestations. Similarly, biological therapy (rituximab) was exclusively used in patients with lung involvement.

Systemic glucocorticoid therapy (methylprednisolone) was administered to all 24 patients (100%). None of the patients were on steroid monotherapy, all received some form of immunosuppressive treatment or IVIG. Initial steroid-sparing agents typically included methotrexate or azathioprine. Subsequent therapy selection was driven by the extent of extramuscular involvement, particularly the presence of ILD. The most frequently used second-line agent was cyclophosphamide (n = 11, 45.8%), followed by methotrexate (n = 9, 37.5%) (Figure 2). Other conventional therapies included azathioprine (25.0%), cyclosporine (25.0%), and hydroxychloroquine (25.0%).

In severe or refractory cases, particularly those with pulmonary involvement, advanced therapies were initiated. Mycophenolate mofetil and intravenous immunoglobulin (IVIG) were each administered to 4 patients (16.7%), while JAK inhibitor therapy (tofacitinib) was used in 4 cases (16.7%). Rituximab was utilized in 3 cases (12.5%), and plasma exchange was performed in 2 patients (8.3%). The median follow-up duration from the time of initial clinical diagnosis was 72.0 months (range: <1 to 336 months). Over the study period the cumulative all-cause mortality rate was 12.5% (n = 3); however, no deaths were directly attributable to IIM-related complications. One patient (67 years old) died of sudden cardiac death; notably, this patient had no evidence of ILD or specific cardiac involvement. A second patient (81 years old) succumbed to the progression of metastatic breast cancer. The third death (72 years old) was caused by hypovolemic shock secondary to retroperitoneal hemorrhage.

## 4. Discussion

Epidemiological data on anti-MDA5 positive myositis vary significantly across different geographical regions. While Japanese and US studies report prevalence rates of 11% and 13% among DM patients, respectively [12,13], European data suggest a much lower frequency (<2%) [14]. In our Hungarian cohort, the prevalence was 3.23%, aligning more closely with European trends but suggesting a potentially higher detection rate in specialized centers.

A striking finding in our cohort was the marked dissociation between systemic inflammatory markers and muscle enzymes. The majority of patients (70.8%) presented with low or normal CK levels, a hallmark of the anti-MDA5 phenotype that frequently contributes to diagnostic delays. Crucially, we observed a similar dissociation regarding CRP: levels remained remarkably low (median 4.24 mg/L) even in patients with active ILD. This suggests that CRP is an unreliable marker for monitoring disease activity in anti-MDA5 dermatomyositis. In contrast, LDH was elevated in 91.7% of patients. Consequently, we propose that LDH may serve as a more sensitive indicator of underlying disease activity and should be prioritized in clinical monitoring.

The clinical presentation of anti-MDA5 positive patients is notably heterogeneous. While US cohorts often present with milder muscle symptoms and arthritis, East Asian populations are characterized by a high incidence of clinically amyopathic dermatomyositis (CADM) and RP-ILD [15]. Recent classifications propose three phenotypes: a “rheumatologic” group (arthritis-dominant), an “RP-ILD” group (high mortality), and an “intermediate” group (skin/muscle involvement) [5,16]. As summarized in Table 2, our cohort showed a mixed distribution. Interestingly, male patients were predominantly found in the intermediate group, while gastrointestinal involvement, particularly dysphagia, appeared more frequent in the “RP-ILD” phenotype in our observation. Assignment of patients to these specific phenotypes was performed retrospectively based on expert clinical judgment, evaluating the predominant clinical features recorded during the disease course.

Our findings highlight distinct geographical variations in the clinical expression and prognosis of anti-MDA5 positive myositis. Established literature indicates that patients with anti-MDA5 positive DM are highly prone to developing ILD, with reported prevalence rates ranging from 50% to 100% [17]. Consequently, anti-MDA5 DM is historically associated with a poor prognosis driven by a high incidence of RP-ILD. For instance, a large cohort study from China reported that 54% of anti-MDA5 positive patients presented with RP-ILD [18]. Furthermore, another Chinese study highlighted the critical need for early prognosis evaluation, noting that 47% of patients succumbed to respiratory failure due to RP-ILD within one year of diagnosis [19,20]. In contrast, the burden of RP-ILD appears less pronounced in Caucasian populations, where 38–73% of patients develop ILD, but only 20–57% exhibit the rapidly progressive form [13,21,22,23]. Cavagna et al. investigated a non-Asian population in 2022. They found that the frequency of ILD and RP-ILD was 78% and 21.5%, respectively [24].

Our Hungarian cohort aligns closely with the patterns observed in other European studies, where the disease is often characterized by a “vasculopathic” or articular dominance rather than fulminant respiratory failure. In our cohort, the observed survival rate suggests the observation that the “Asian-derived” mortality risk profile may not be directly applicable to Central European populations.

These phenotypic discrepancies are likely underpinned by immunogenetic differences. Yang et al. identified HLA-DQA1*06:01 as a novel genetic and prognostic factor for anti-MDA5 positive DM in Asian populations, potentially serving as a biomarker for early diagnosis [25]. Similarly, Chen et al. observed significantly higher frequencies of DRB1*04:01 and *12:02 in anti-MDA5 positive patients with DM-ILD [26]. Conversely, Caucasian cohorts are more frequently associated with HLA-DRB1*03:01 allele [27] that may confer protection against the most severe pulmonary manifestations. Although genetic testing was not performed in our study, the favorable outcomes observed strongly suggest that ethnicity plays a decisive role in disease behavior, necessitating region-specific management algorithms rather than a uniform global approach.

Interstitial lung disease warrants distinct consideration, given its frequency (58.3%) and prognostic weight. Consistent with previous findings [2], the dominant radiological patterns were OP and NSIP. Our results reinforce the critical role of anti-Ro52 as an independent risk factor for pulmonary involvement [28,29]. We identified a strong predictive association: 71.4% of ILD patients were anti-Ro52 positive, whereas only 10% of non-ILD patients carried this antibody. Statistical analysis confirmed this link with a high odds ratio (22.5). Although the wide confidence interval (95% CI: 2.10–240.48) reflects the limitations of the sample size, the lower bound >1 suggests a relevant association. However, the strength of anti-Ro52 as a risk factor should be interpreted with caution. Furthermore, both cases of RP-ILD in our cohort were anti-Ro52 positive, supporting the hypothesis that this co-occurring antibody drives a more severe pulmonary phenotype [30,31]. In contrast, pulmonary involvement in patients positive for anti-PL-7 or anti-PL-12 did not manifest as RP-ILD. Beyond anti-Ro52, we noted two other distinct associations. First, anti-SAE co-occurred with anti-MDA5 in 12.5% of cases, all presenting with extensive cutaneous involvement (erythema, ulcers, facial oedema). Second, the co-existence of dermatomyositis and primary immunodeficiency (PID) in two cases highlights a complex pathophysiological link between autoimmunity and inborn errors of immunity, potentially driven by dysregulated interferon pathways.

Therapeutic management followed an aggressive approach adapted from recent expert recommendations. The therapeutic algorithms utilized in our cohort were consistent with those reported in other international cohorts. Anti-MDA5 positivity is historically considered a predictor of poor prognosis, particularly in East Asian cohorts where RP-ILD-associated mortality is high [32,33,34]. In sharp contrast, our study demonstrated excellent survival outcomes. We observed 100% disease-specific survival over the 6-year study period; deaths were unrelated to autoimmune complications. This favorable outcome is likely attributable to two factors: a potentially less fulminant biological phenotype in Central European Caucasians compared to Asian populations, and the implementation of early, aggressive multimodal therapy (combining high-dose corticosteroids with cyclophosphamide, calcineurin inhibitors, or JAK inhibitors) upon diagnosis. In the future to optimize treatment and improve patient safety, vaccination status should be updated and completed whenever possible prior to treatment initiation for anti-MDA5 positive dermatomyositis.

Our study has certain limitations inherent to its design. First, the retrospective nature and the relatively small sample size, although derived from a multicenter collaboration, limit the generalizability of our findings. However, considering the ultra-rare nature of anti-MDA5 positive dermatomyositis, achieving a large cohort in a single European country remains highly challenging without international registries. Specifically, the wide confidence intervals observed regarding the association between anti-Ro52 and ILD necessitate a cautious interpretation of the statistical strength of this predictor. Second, since data were collected from tertiary referral centers, a referral bias cannot be excluded, as milder cases might have been managed at local facilities. Furthermore, as this was a retrospective clinical study reflecting real-world diagnostic protocols, routine genetic typing (e.g., HLA typing) was not available. Future prospective studies incorporating detailed genomic data are warranted to definitively confirm the underlying immunogenetic mechanisms hypothesized in this cohort. Finally, while our survival data is excellent, we must acknowledge a potential survival bias affecting this figure: the 100% disease-specific survival rate might reflect the exclusion of rapidly fatal cases occurring before referral to tertiary centers, which would skew the overall mortality profile.

## 5. Conclusions

To the best of our knowledge, this is the first multicenter retrospective study characterizing anti-MDA5 positive patients in Hungary. Our findings confirm that this entity represents a heterogeneous disease group, particularly regarding the presentation and progression of interstitial lung disease (ILD) and rapidly progressive ILD (RP-ILD). The presence of anti-Ro52 emerged as a notable factor, serving as a potential predictor for pulmonary involvement. A crucial finding of our study is that no ILD-associated mortality occurred in our cohort.

Our results demonstrate that early identification and the prompt initiation of intensive, multi-modal therapeutic approaches are critical, as they can lead to excellent survival outcomes in European cohorts, compared with historical data based on Asian cohorts. From a clinical perspective, the characteristic dissociation between low CK/CRP levels and elevated LDH underscores the clinical utility of LDH as a superior marker for monitoring disease activity. While our results are encouraging, further large-scale, prospective studies are needed to refine screening protocols and establish standardized management strategies for this complex clinical phenotype.

## Figures and Tables

**Table 1:** Demographic, clinical, serological, and laboratory characteristics of the anti-MDA5 positive cohort (N = 24).

Variable	Value (N = 24)
**Demographics**	
Age at diagnosis, median (range)	49.5 (24–81) years
Gender, female:male, n (%)	15:9 (62.5%:37.5%)
**Clinical Subgroups, n (%)**	
Dermatomyositis (DM)	18 (75.0%)
Amyopathic DM (ADM)	3 (12.5%)
Polymyositis (PM)	3 (12.5%)
**General and Systemic Symptoms, n (%)**	
Fever	5 (20.8%)
Raynaud’s phenomenon	5 (20.8%)
Mechanic’s hands	4 (16.7%)
Subcutaneous calcinosis	1 (4.2%)
Recurrent malignancy (breast cancer)	1 (4.2%)
Organ Involvement, n (%)	
Muscle weakness (all non-ADM patients)	21 (87.5%)
Cutaneous symptoms (all non-PM patients)	21 (87.5%)
Arthritis/Arthralgia	18 (75.0%)
Interstitial Lung Disease (ILD)	14 (58.3%)
Dysphagia	5 (20.8%)
Myocarditis	3 (12.5%)
**Laboratory Parameters (at diagnosis)**	
CK level, median (range)	193.5 (21–3463) U/L
CK < 500 U/L, n (%)	17 (70.8%)
LDH level, median (range)	377.0 (233–1538) U/L
Elevated LDH (>250 U/L), n (%)	22 (91.7%)
AST level, median (range)	36.0 (18.0–78.0) U/L
ALT level, median (range)	29.0 (12.0–86.0) U/L
CRP level, median (range)	4.24 (0.66–33.0) mg/L
**Serology, n (%)**	
Anti-Ro52 positivity	11 (45.8%)
Anti-SAE positivity	3 (12.5%)
Anti-NXP2 positivity	2 (8.3%)
Anti-PL-7 positivity	1 (4.2%)
Anti-PL-12 positivity	2 (8.3%)
Anti-SRP positivity	1 (4.2%)
ANA positivity	8 (33.3%)
**Outcomes, n (%)**	
Overall mortality	3 (12.5%)
Disease-related mortality	0 (0%)

(Abbreviations: ALT: alanine aminotransferase, ANA: antinuclear antibody, AST: aspartate aminotransferase, CK: creatine kinase, CRP: C-reactive protein, LDH: lactate dehydrogenase).

**Table 2:** Characteristics of clinical phenotypes in anti-MDA5 positive patients: A comparison of our cohort with literature data [14].

Patients with Anti-MDA5 Antibodies			
	Phenotype 1	Phenotype 2	Phenotype 3
According to Nombel A et al. 2021 [16]			
Frequency	≈20%	≈50%	≈30%
Gender	women > men	men > women	women > men
Main clinical symptoms	arthralgia/arthritis	skin vasculopathies severe proximal muscle weakness	RP-ILD mechanics hand
Our results			
Frequency	4 patients (16.67%)	18 patients (75%)	2 patients (8.33%)
Gender	women > men (4:0)	women ≈ men (10:8)	women = men (1:1)
Main clinical characteristics	RA-like joint symptoms	DM-specific skin lesions muscle weakness dysphagia (n = 5) higher CK at diagnosis Co-existence of anti-SAE (n = 3)	DM RP-ILD

(Abbreviations: CK: creatine kinase, DM: dermatomyositis, RA: rheumatoid arthritis, RP-ILD: rapidly progressive interstitial lung disease, SAE: small ubiquitin-like modifier activating enzyme).

## Data Availability

The data presented in this study are available on request from the corresponding author.

## Abbreviations

The following abbreviations are used in this manuscript:

ACR/EULAR	American College of Rheumatology/European Alliance of Associations for Rheumatology
ADM	Amyopathic dermatomyositis
ANA	Antinuclear antibody
Anti-MDA5	Anti-melanoma differentiation-associated gene 5
CADM	Clinically amyopathic dermatomyositis
CI	Confidence interval
CK	Creatine kinase
CRP	C-reactive protein
DLCO	Diffusing capacity of the lung for carbon monoxide
DM	Dermatomyositis
FVC	Forced vital capacity
HLA	Human leukocyte antigen
HRCT	High-resolution computed tomography
IIMs	Idiopathic inflammatory myopathies
ILD	Interstitial lung disease
IMACS	International Myositis Assessment and Clinical Studies Group
IVIG	Intravenous immunoglobulin
JAK	Janus kinase
LDH	Lactate dehydrogenase
MAAs	Myositis-associated antibodies
MSAs	Myositis-specific antibodies
NSIP	Non-specific interstitial pneumonia
OP	Organizing pneumonia
OR	Odds ratio
PID	Primary immunodeficiency
PM	Polymyositis
RA	Rheumatoid arthritis
RP-ILD	Rapidly progressive interstitial lung disease
SAE	Small ubiquitin-like modifier activating enzyme
SSc	Systemic sclerosis
UIP	Usual interstitial pneumonia
